# Predicting Anxiety in Children Aged 2–6 During Preoperative Anesthesia Consultation—A Prospective Observational Study

**DOI:** 10.1002/pan.70101

**Published:** 2025-12-09

**Authors:** Armin Sablewski, Charlotte Neitzel, Maximilian Grosser, Katarina Krebs, Anna Karstensen, Alina Balandin, Helene Selpien, Tobias Becher

**Affiliations:** ^1^ Department of Anesthesiology and Intensive Care Medicine University Hospital S‐H Kiel Germany

**Keywords:** anxiety prediction, mYPAS, pediatric anesthesia, preoperative anxiety, preoperative assessment

## Abstract

**Background:**

Preoperative Anxiety in Young Children Is Common and Can Lead to Adverse Outcomes. In Clinical Routine, Anesthesiologists Must Often Predict Anxiety Based on Limited Interaction.

**Aims:**

This study aimed to evaluate the accuracy of early anxiety predictions and to identify early predictors of heightened anxiety at anesthesia induction.

**Methods:**

In this prospective observational study, anesthesiologists and parents of children aged 2–6 years undergoing elective procedures were asked during the preoperative consultation to predict the child's anxiety at anesthesia induction using the visual analog scale (VAS). These predictions were compared to observed anxiety during induction, measured with the Modified Yale Preoperative Anxiety Scale—Short Form (mYPAS‐SF). Prediction accuracy was assessed using Spearman's correlation (*r*_s_) and receiver operating characteristics (ROC) analysis. Potential predictors of significant anxiety defined as a mYPAS‐SF > 30 were analyzed.

**Results:**

A total of 92 prediction sets were analyzed. Correlation between predicted and observed anxiety was weak for parents (*r*_s_ = 0.220, 95% CI 0.01–0.41) and very weak for anesthesiologists (*r*_s_ = 0.106, 95% CI −0.11–0.31). Predictive performance was limited for parents (AUC = 0.643) and negligible for anesthesiologists (AUC = 0.517). Children who responded positively to a greeting (‘high‐five’) during consultation showed significantly lower anxiety during anesthesia induction (median mYPAS‐SF score 34.4 [22.9–65.1] vs. 75.0 [45.8–90.6], *p* < 0.001). Significant anxiety was also associated with younger age of both children and parents, migration background, and inhalational induction.

**Conclusions:**

Anxiety at induction remains difficult to predict during preoperative consultation. While parents performed slightly better than anesthesiologists, both lack sufficient precision. Simple behavioral cues, such as a response to a greeting, may help identify at‐risk children early. Future strategies should involve children and parents in individualized anxiety management.

**Trial Registration:**

German Clinical Trials Registry, registration number: DRKS00035033

## 1 Introduction

Preoperative anxiety affects up to 70% of young children and is associated with adverse outcomes including increased pain, emergence delirium, behavioral disturbances, and delayed recovery [1, 2]. Consequently, anxiety management is a central focus of pediatric anesthesia quality standards and a recognized priority for patients and their families [3, 4].

In clinical practice, anxiety is often assessed using observational tools such as the Modified Yale Preoperative Anxiety Scale (mYPAS) and its short form (mYPAS‐SF), both of which have been validated for use in children in perioperative context [5]. Visual analog scales (VAS) are also frequently used due to their simplicity and broad applicability [6, 7]. The induction compliance checklist (ICC) provides additional information on behavioral distress during mask induction [8, 9].

Multiple factors are associated with heightened preoperative anxiety, including younger age, internalizing traits, parental anxiety, language or cultural barriers, and previous negative hospital experiences [10, 11, 12, 13]. In addition to these patient‐related factors, several psychological instruments have been developed to assess child and parental traits. These include the emotionality, activity, sociability, and impulsivity (EASI) inventory, the integrative child temperament inventory (ICTI), as well as parental and children's trait anxiety using the state–trait anxiety inventory (STAI), and some of these traits are associated with increased preoperative anxiety in children [14, 15, 16, 17, 18]. However, these tools remain largely theoretical and have not yet been translated into structured risk assessment in everyday clinical practice.

Despite these known factors, anesthesiologists often continue to rely on clinical intuition when determining whether and how to initiate preparatory strategies, including the use of premedication [19]. Another challenge for predicting children's perioperative distress lies in the time gap between preoperative consultation and surgery: consultations are typically conducted in advance, when the child is calm and in a familiar environment. Yet anxiety often increases as surgery approaches and may peak at induction [20], making early predictions particularly unreliable and initial clinical impressions may therefore be misleading. On the other hand, early consultations may also provide an opportunity to identify known risk factors and initiate targeted interventions to reduce perioperative anxiety. However, how this gap is currently addressed in clinical practice, and how effectively parents and clinicians navigate it, remains unclear.

This prospective observational study aimed to evaluate whether parents and anesthesiologists can reliably anticipate children's anxiety at induction based on assessments during routine preoperative consultations. We hypothesized that parents and anesthesiologists may be able to anticipate children's perioperative anxiety during these consultations. In addition, we aimed to identify simple behavioral and contextual predictors that could serve as pragmatic tools to facilitate early recognition of children at risk for heightened anxiety.

## 2 Methods

### 2.1 Study Design

This prospective monocentric observational trial was registered at the German Clinical Trials Registry (date of first trial registration 06/09/2024, registration number).

(DRKS00035033, https://drks.de/register/en/trial/DRKS00035033/preview) and was approved by the local Institutional Review Board (Ethics Committee of Christian‐ Albrechts‐University of Kiel, Germany, ethics approval number D508/24). This observational cross‐sectional cohort study was performed in accordance with the Equator Network STROBE guidelines [21], relevant regulatory standards, and the Declaration of Helsinki [22].

### 2.2 Setting and Participants

The study was conducted at the University Medical Center Schleswig‐Holstein (UKSH), Kiel Campus, Germany, between September 2024 and April 2025. Screening was performed daily on weekdays by the study team at the preoperative anesthesia clinic. The number of screened and included patients, along with reasons for non‐inclusion, was documented anonymously in a pre‐screening log.

Children aged 2–6 years scheduled for elective procedures (surgery or diagnostic interventions) requiring general anesthesia were eligible. Exclusion criteria were language barriers (requiring the use of an interpreter) for parents or legal guardians, as well as previous participation in this study. Written informed consent was obtained from the parents or legal guardians.

### 2.3 Data Sources/Measurement

**FIGURE 1:**
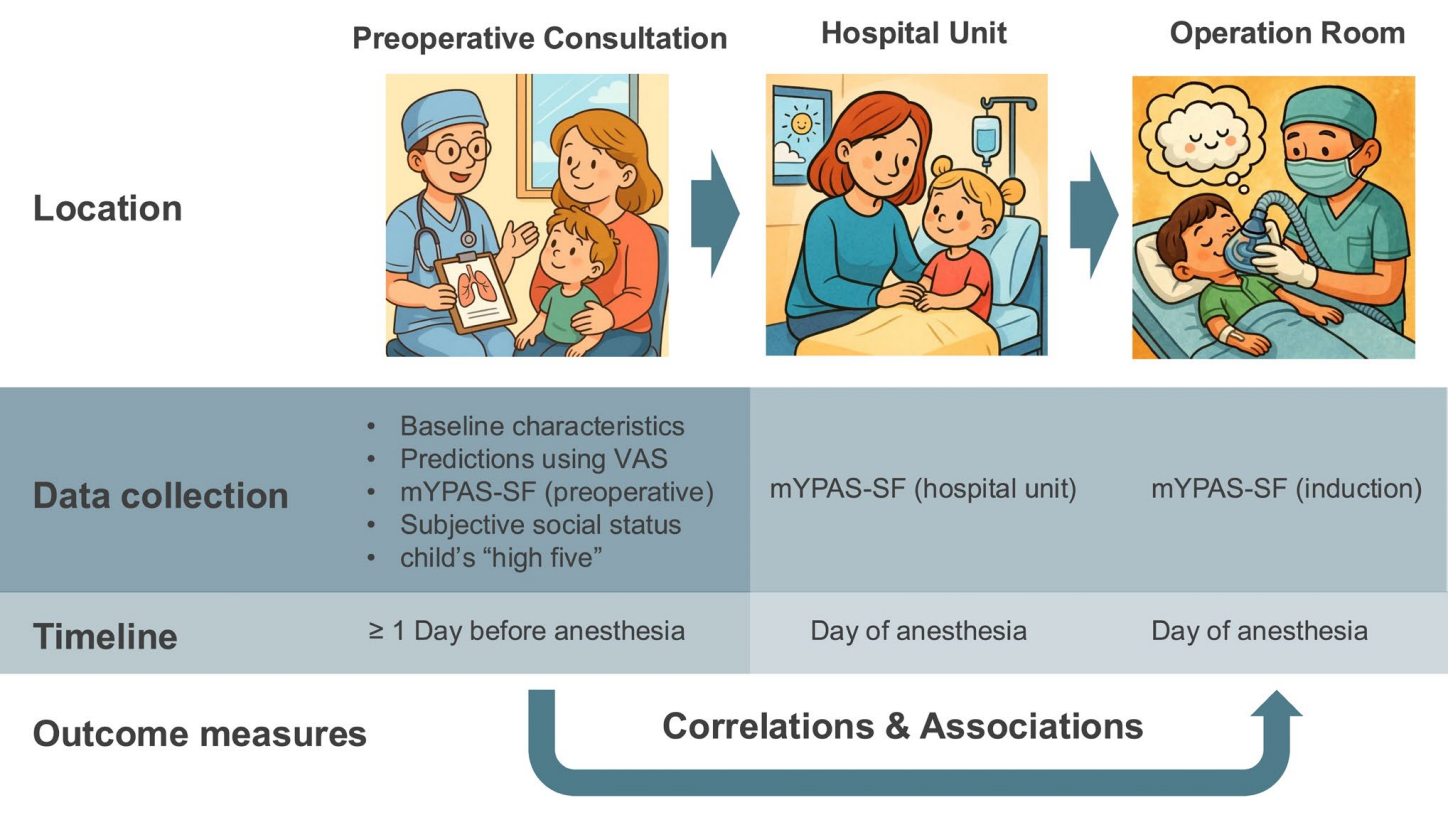
Schematic overview of data collection points and outcome assessment across the three clinical phases. mYPAS‐SF, Short Form of the Modified Yale Preoperative Anxiety Scale.VAS, Visual Analog Scale.

All measurements were performed at three time points: during the preoperative consultation, on the hospital unit, and during induction of anesthesia (defined as the start of preoxygenation). Figure 1 provides an illustrated overview of the study design.

### 2.4 Setting and Assessments During Preoperative Consultation

At the beginning of the preoperative consultation, children were greeted by a member of the study team and invited to respond with a “high‐five” and their reaction (yes or no) to this greeting was documented. After informed consent had been obtained, demographic data and baseline measures were collected along with information on the child's and parental native languages and migration status.

To estimate children's anticipated anxiety, a visual analogue scale (VAS) was completed by both parents and anesthesiologists during the anesthesia consultation. The VAS consisted of a 100 mm horizontal line anchored by two pictorial representations of behavioral extremes. Scores ranged from 0 to 100, with higher scores indicating higher anxiety levels. VAS scores (0–100) were rounded to a scale from 0 to 10 for analysis. The VAS is commonly used to measure subjective states like general anxiety, preoperative anxiety, and pain [7, 23, 24]. In this study, parents and anesthesiologists marked a line to indicate the child's anticipated anxiety in the operating room just before the induction of anesthesia, as described previously [25].

Additionally, parental anxiety was self‐rated using the VAS after the preoperative consultation, with scores ≥ 4 indicating clinically relevant anxiety.

Children's baseline anxiety was assessed using the mYPAS‐SF. The mYPAS‐SF is an observational measure of preoperative anxiety consisting of 18 items in four domains (activity, emotional expressivity, state of arousal, and vocalization). The mYPAS‐SF total score ranges from 22.9 to 100, with higher scores indicating greater anxiety [5]. A mYPAS‐SF score > 30 was used to define the presence of clinically significant anxiety as described in previous publications [26]. New raters underwent training with experienced pediatric anesthesiologists and jointly assessed children to ensure a consistent understanding and application of the mYPAS‐SF scoring system. Once their scoring was deemed consistent with the experienced raters, they were allowed to perform independent assessments. Whenever possible, the same two raters assessed the mYPAS‐SF. Interrater reliability was evaluated using the intraclass correlation coefficient (ICC) for all cases in which both raters were present.

The German version of the MacArthur Scale was used to measure subjective socioeconomic status (SES) during preoperative consultation. Parents were asked to place themselves on a 10‐rung ladder (ranging from 1 to 10), with higher rungs indicating higher income, education, and job status relative to others in Germany [27, 28].

Parents were asked to rate their perception of the planned medical procedure using a 3‐point Likert scale (small, medium, large), which was included as an exploratory measure to capture subjective parental appraisals of the planned procedure. Parents were asked to provide two types of subjective ratings: a perceived procedural extent (“How would you rate the extent of the procedure?”) and a perceived personal significance (“How significant is the procedure for you personally?”).

Additionally, the study team independently categorized all medical procedures as small (e.g., MRI scans, hernia surgery), medium (e.g., osteosynthesis), or large (e.g., exploratory laparotomy) based on institutional pediatric anesthesia standards and in line with previously published classifications [29]. The classification considered expected invasiveness, duration, and expected postoperative discomfort. The distribution of these procedural categories was summarized descriptively.

In line with institutional standards, midazolam was prescribed at a dose of 0.5 mg/kg, with exceptions made for procedures performed in the ambulatory surgery unit (day case) or prior to MRI scans. All attending anesthesiologists were familiar with the hospital's protocols. Deviations from institutional standard recommendations were permitted at the discretion of the anesthesiologist, based on individual clinical judgment. Decisions regarding the administration of premedication were documented.

### 2.5 Setting and Assessments on the Day of Surgery

On the day of surgery, children's anxiety was assessed on the hospital unit using the mYPAS‐SF prior to midazolam administration.

A topical anesthetic cream with lidocaine and prilocaine (EMLA, Aspen Pharmacare, Durban, South Africa) was applied to an appropriate skin area 30–60 min before transfer to the operating room or examination area. Parents did not accompany their children during anesthesia induction, remaining instead in the holding area except for procedures in the ambulatory surgery unit (day cases) or for MRI examinations.

All children adhered to the perioperative fasting guidelines established by the European Society of Anesthesiology and Intensive Care [30]. Anesthesia preparation, including the placement of electrocardiography electrodes, a pulse oximeter, and a blood pressure cuff, was performed in the operating room. Anesthesia was induced intravenously (IV) whenever possible. If IV line placement was not tolerated or unsuccessful, inhalation induction was performed instead. For IV induction, a peripheral IV line was inserted in the area pretreated with a topical local anesthetic, followed by preoxygenation and administration of propofol (2–5 mg/kg IV) and, if appropriate, an opioid (sufentanil 0.25–0.5 mcg/kg IV or remifentanil 0.2–0.4mcg/kg/min). For inhalation induction, sevoflurane in a mixture of oxygen and air was administered via face mask, followed by IV line placement after loss of consciousness. For both settings, the mYPAS‐SF was reassessed during preoxygenation.

Additionally, behavioral distress was evaluated with the Induction Compliance Checklist (ICC). The ICC represents the sum of 10 negative behavioral categories evaluated during induction, with higher scores indicating lower behavioral compliance [8]. Anesthesia induction was performed by board‐certified anesthesiologists or under their supervision, ensuring a professional, child‐friendly, and empathetic approach within a supportive environment.

Details regarding the anesthesia induction setting were documented, including the use of pharmacological and non‐pharmacological interventions, mode of induction (inhalation or intravenous), and the experience level of the anesthesia team.

### 2.6 Statistical Methods

The primary analysis focused on Spearman's correlation (*r*_s_) between anesthesiologists' anxiety predictions, assessed using the VAS during the preoperative consultation, and children's observed anxiety at anesthesia induction, measured with mYPAS‐SF. Based on prior research suggesting a correlation of 0.3, we estimated the required sample size using G*Power (version 3.1.9.6) [25]. Assuming a two‐sided significance level of 0.05 and a power of 0.80, a sample size of 89 prediction pairs was required. To compensate for potential dropouts, we increased the target recruitment by 5%, resulting in a final sample size of 92 patients. The same sample size calculation was applied to the analysis of parental anxiety predictions.

In the descriptive analysis, baseline characteristics were presented as mean (±SD) for normally distributed continuous variables, and as median and interquartile range [25th–75th percentile] for non‐normally distributed continuous variables. The Kolmogorov–Smirnov test was used to evaluate the normality of data distribution. Categorical variables were reported as absolute and relative frequencies. To assess potential associations between predictor and outcome variables, univariate analyses were performed. Categorical variables were analyzed using Fisher's exact test or chi‐square test, as appropriate. Continuous variables were compared using the Mann–Whitney *U* test for non‐normally distributed data. For group comparisons of non‐parametric data, the Kruskal–Wallis test was applied. All tests were two‐sided, with *p*‐values ≤ 0.05 considered statistically significant.

Additionally, the predictive performance for clinically significant anxiety during anesthesia induction (defined as mYPAS‐SF > 30) was assessed by calculating the area under the receiver operating characteristic curve (AUC).

To account for potential confounding, we first performed univariable logistic regression to examine the crude association between VAS predictions and clinically significant anxiety (mYPAS‐SF > 30). We then performed multivariable logistic regression with clinically significant anxiety as the dependent variable and the respective VAS predictions as the main independent variables. The model was adjusted for age, premedication (yes/no), parental presence (yes/no), and mode of induction (intravenous vs. inhalational). Adjusted odds ratios (aOR) with 95% confidence intervals (95% CIs) were reported.

Subgroup analyses stratified by premedication, parental presence, and mode of induction were conducted to assess the robustness of the association under different clinical conditions. Within each subgroup, the association between VAS ratings and observed anxiety was examined using again Spearman's correlation (*r*_s_).

We calculated the intraclass correlation coefficient (ICC) among patients rated by the same two raters to assess interrater reliability. ICC was computed using a two‐way random‐effects model with absolute agreement for single measures (model = “twoway”, type = “agreement”, unit = “single”) in the irr package in R [31].

Analyses and visualizations were performed using GraphPad Prism (GraphPad Software, La Jolla, CA, USA), Microsoft Excel (Office 2019, Microsoft Corporation, Redmond, WA, USA), and R version 4.5.1 (R Foundation for Statistical Computing, Vienna, Austria). Images were AI‐generated (DALL·E, OpenAI, ChatGPT; June 2025 version, USA) without the use of any original or copyrighted material.

## 3 Results

### 3.1 Baseline Characteristics

**FIGURE 2:**
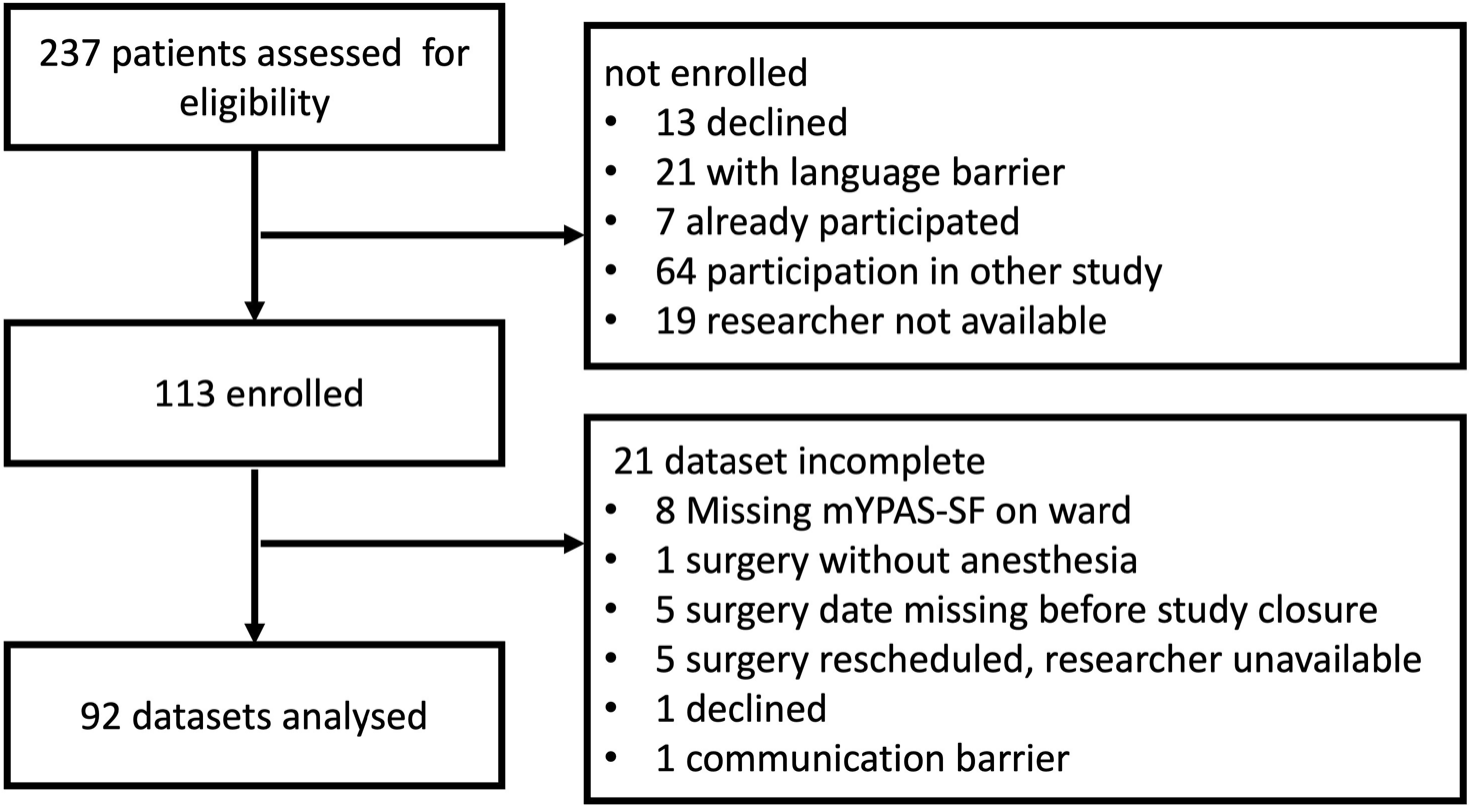
Participant workflow diagram for the full‐analysis set. mYPAS‐SF, Short Form of the Modified Yale Preoperative Anxiety Scale.

A total of 237 patients aged 2–6 years met the inclusion criteria, of whom 92 were included in the study (full analysis set, see Figure 2) thus meeting the predefined sample size of 89 required for adequate statistical power. Unless stated otherwise, all data and results refer to this primary analysis population. The baseline characteristics of the included patients are presented in Table 1. The trial concluded as planned in April 2025, having successfully recruited the targeted number of participants.

**TABLE 1:** Characteristics of children and accompanying parent(s).

Characteristic	Total (*N* = 92)
Age, years	4.2 [2.9–5.7]
Male	50 (54.3%)
Female	42 (45.7%)
Weight, kg	17 [15–20]
Height, cm	106 [96–114]
ASA	
I	57 (62.0%)
II	15 (16.3%)
III	20 (21.7%)
Reason for anesthesia	
Surgery	61 (66.3%)
Examination	31 (33.7%)
Treatment setting	
Outpatient (daycase)	56 (60.9%)
Hospitalized	36 (39.1%)
First hospital experience, *n* (%)	71 (77.2%)
First anesthesia experience, *n* (%)	65 (70.7%)
Department	
General Surgery	26 (28.3%)
Pediatrics (MRI scans)	26 (28.3%)
ENT	22 (23.9%)
Orthopedic surgery	8 (8.7%)
Oral and Maxillofacial Surgery (OMFS)	5 (5.4%)
Urology	3 (3.3%)
Ophthalmology	2 (2.2%)
Sociodemographic and Family Characteristics	
Child accompanied by	
Mother	75 (81.5%)
Father	45 (48.9%)
Both	28 (30.4%)
Parents' age	36.2 ± 7.3
Migration background child (yes)	19 (20.7%)
Children's native language (German)	77 (83.7%)
Subjective social status [1–10]	
Mother	6.2 ± 1.9
Father	6.4 ± 1.7

Abbreviations: ASA, American Society of Anesthesiologists Physical Status Classification System; ENT, Ear, Nose, and Throat (Otorhinolaryngology); OMFS, Oral and Maxillofacial Surgery.

### 3.2 Preoperative Consultation Setting

When entering the consultation room, 50 children engaged in a high‐five or handshake (54.3%). The distribution of reactions to a high‐five across age groups is presented in the supplement (Figure S1).

The professional experience of the anesthesiologists for the preoperative consultation ranged from 0 to 17 years, with a median of 3.5 [1–5] years. Among them, 17 (18.5%) were board‐certified specialists.

In terms of premedication, midazolam was prescribed in 62 instances (67.4%). For midazolam, the median prescribed dose was 7 [0–9] mg or 0.4 [0–0.5] mg/kg of body weight. The reasons for and against the prescription of anxiolytic medication are summarized in Table 2.

**TABLE 2:** Reasons for or against premedication.

Reason for premedication	Total *N* = 102	Reason against premedication	Total *N* = 38
Child's anxiety	25 (24.5)	Due to the setting‐specific routine	13 (34.2)
Medical history	20 (19.6)	Assumed not anxious	10 (26.3)
Tendency to premedicate	18 (17.6)	Due to the surgery	8 (21.1)
Parental's wish	15 (14.7)	Medical history/comorbidities	6 (15.8)
Own experience/gut feeling	10 (9.8)	Parental refuse	1 (2.6)
Child's wish	6 (5.9)	Tendency to avoid	0
Parental anxiety	6 (5.9)		
Others	2 (2.0)		

Multiple answers were possible. Other: 1× due to specific medical condition, 1× due to specific surgery context.

The median mYPAS‐SF score for children's anxiety during the preoperative consultation was 22.9 [22.9–32.8], and 25 children (27.2%) showed significant anxiety (mYPAS‐SF > 30). The intraclass correlation coefficient for mYPAS‐SF scoring was 1.0 (*n* = 6).

Based on the perceived procedural extent, parents rated the procedure as small in 62 cases (67.4%), medium in 22 (23.9%), and large in 8 (8.7%). In contrast, ratings of personal significance yielded higher severity estimates, with 27 (29.3%) rated as small, 21 (22.8%) as medium, and 44 (47.8%) as large. According to the study team's objective classification, procedures were categorized as small in 44 cases (47.8%), medium in 45 (48.9%), and large in 3 (3.3%). Both the difference between perceived procedural extent and personal significance (*χ* ^2^(2) = 74.9, *p* < 0.001) and the difference between perceived and objective extent (*χ* ^2^(2) = 27.5, *p* < 0.001) were statistically significant. The observed anxiety at induction of anesthesia as measured with the mYPAS‐SF did not differ significantly across any of these classifications (all *p* > 0.05, Kruskal–Wallis tests). Parental anxiety, assessed immediately after the consultation, had a median VAS score of 5 [0–6]. 54 parents (58.7%) reported a VAS score ≥ 4, indicating clinically relevant concern.

### 3.3 Hospital Unit Setting

The median mYPAS‐SF score in the hospital unit prior to anti‐anxiety interventions was 29.2 [22.9–43.2], and 38 children (41.3%) had significant anxiety (mYPAS‐SF > 30). The intraclass correlation coefficient for mYPAS‐SF scoring was 0.92 (*n* = 15).

### 3.4 Anesthesia Induction Setting

The median professional experience of anesthesiologists for the induction of anesthesia was 8 years [5–15]. Parental presence during induction was reported in 66 instances (71.7%). Non‐pharmacological interventions were applied in 79 instances (85.9%). Table S1 provides a tabular overview of the non‐pharmacological interventions used. 38 patients received midazolam with a median dose of 7.75 mg [6–10] or 0.5 mg/kg [0.4–0.5]. Prior to anesthesia induction, 61 patients (66.8%) showed no sedative effect, 25 (27.2%) were calm, and 6 (6.5%) were asleep. Regarding the mode of anesthesia induction, 45 patients (48.9%) underwent inhalational induction, while 47 patients (51.1%) received intravenous induction.

### 3.5 Accuracy of Anxiety Prediction

The median mYPAS‐SF score during anesthesia induction was 49.5 [29.2–84.9], and 64 children (69.6%) showed significant anxiety. The intraclass correlation coefficient for mYPAS‐SF scoring was 0.93 (*n* = 16).

Parents predicted their children's anxiety with a median VAS score of 7 [4–8], while anesthesiologists provided a median score of 5 [2–7] (*p* < 0.001). Agreement between predicted and observed anxiety yielded values that were weak for parents (*r*_s_ = 0.220; 95% CI 0.01–0.41) and negligible for anesthesiologists (*r*_s_ = 0.106; 95% CI −0.11–0.31). The AUC for predicting clinically significant anxiety during anesthesia induction was 0.643 (95% CI: 0.528–0.758, SE: 0.0587, *p* = 0.029) for parents and 0.517 (95% CI: 0.395–0.639, SE: 0.0623, *p* = 0.799) for anesthesiologists.

The median ICC was 7 [4–8], with 20 children (24.7%) being fully compliant (ICC = 0), while 61 (75.3%) showed some degree of behavioral distress (ICC > 0). Data for the ICC were missing in 11 instances. The ICC score showed a strong correlation with observed anxiety at induction (mYPAS‐SF; *r*_s_ = 0.84; 95% CI: 0.76–0.90; *p* < 0.001). There was no significant correlation between the ICC score and anxiety predictions by parents (*r*_s_ = 0.14; 95% CI: −0.09–0.35) or anesthesiologists (*r*_s_ = −0.01; 95% CI: −0.23–0.22).

### 3.6 Predictor Variables for Children's Anxiety

Univariate results are presented in Tables 3 and 4. Among the preoperative consultation parameters, Spearman's correlation showed that a younger age of both children and parents, and a migration background were associated with higher anxiety levels during the induction of anesthesia (see Table 3), while children who responded to “high‐five” showed significantly lower anxiety. Within the anesthesia setting, only the use of inhalational induction was linked to higher anxiety (Table 4).

**TABLE 3:** Univariate associations between continuous predictor variables and the child's anxiety during anesthesia induction.

Predictor variables	*r*_s_ (95% CI) for mYPAS‐SF	*r*_s_ (95% CI) for ICC
Age	−0.40 (−0.56 to −0.20)	−0.35 (−0.54 to 0.14)
Parents age	−0.28 (−0.46 to −0.07)	−0.23 (−0.43 to −0.01)
mYPAS‐SF (preoperative)	0.20 (−0.01 to 0.40)	0.14 (−0.08 to 0.36)
mYPAS‐SF (hospital unit)	0.19 (−0.02 to 0.39)	−0.04 (−0.27 to 0.18)
Parental anxiety	−0.07 (−0.28 to 0.14)	−0.07 (−0.29 to 0.16)
Mother SES	−0.11 (−0.34 to 0.13)	−0.12 (−0.36 to 0.14)
Father SES	0.05 (−0.25 to 0.35)	−0.02 (−0.34 to 0.31)

Abbreviations: ICC, Induction Compliance Checklist; mYPAS‐SF, Short Form of the Modified Yale Preoperative Anxiety Scale; SES, Subjective Socioeconomic Status assessed with the German Version of the MacArthur Scale.

**TABLE 4:** Univariate associations between categorical predictor variables and the child's anxiety during anesthesia induction.

Category	mYPAS‐SF	*p*	ICC	*p*
**Preoperative consultation setting**				
Gender		0.064		0.145
Female (*n* = 42)	65.1 [32.0–89.6]		4 [0–6]	
Male (*n* = 50)	40.6 [22.9–76.3]		1 [1–4]	
Treatment setting		0.840		0.534
Day case (*n* = 56)	47.9 [29.2–87.0]		3 [0–6]	
Hospitalized (*n* = 36)	63.0 [24.5–82.3]		1 [1–4]	
Hospital experience		0.804		0.698
Yes (*n* = 71)	47.9 [29.2–83.3]		2 [0–6]	
No (*n* = 21)	50.0 [26.0–85.4]		2.5 [1–5.75]	
Anesthesia experience		0.672		0.897
Yes (*n* = 66)	48.4 [29.2–86.7]		2.5 [0–6]	
No (*n* = 26)	55.2 [22.9–83.9]		2 [1–5]	
Response to “high‐five”		**< 0.001**		0.029
Yes (*n* = 50)	34.4 [22.9–65.1]		1 [0–4]	
No (*n* = 42)	75.0 [45.8–90.6]		4 [1–6]	
**Child's native language**		0.057		0.069
German (*n* = 77)	47.9 [22.9–81.8]		2 [0.25–5]	
Non‐German (*n* = 15)	77.1 [32.3–100]		5 [1–7]	
**Migration status (child)**		**0.009**		0.031
Yes (*n* = 19)	77.1 [33.3–95.8]		5.5 [1.25–7]	
No (*n* = 73)	45.8 [22.9–79.2]		2 [0–4.5]	
**Anesthesia setting**				
Anesthesia induction		**< 0.001**		0.001
Inhalation (*n* = 45)	77.1 [38.5–93.8]		4 [1.25–6]	
Intravenous (*n* = 47)	35.4 [22.9–62.5]		1 [0–4]	
**Anti‐anxiety intervention**				
Pharmacological		0.335		0.127
Yes (*n* = 38)	42.7 [27.6–79.2]		1.5 [0–4.25]	
No (*n* = 54)	50.0 [29.2–89.6]		4 [1–6]	
Non‐pharmacological		0.825		0.569
Yes (*n* = 80)	49.5 [29.2–83.3]		2 [0.5–6]	
No (*n* = 12)	52.1 [22.9–97.4]		3.5 [0.25–6.5]	

Abbreviations: mYPAS‐SF: Short Form of the Modified Yale Preoperative Anxiety Scale; ICC: Induction Compliance Checklist; IV: intravenous. Bold values indicate statistically significant *p*‐values (≤ 0.05).

Univariate analysis revealed a crude association between parental VAS ratings and clinically significant anxiety (OR 1.15, 95% CI 0.99–1.34), whereas anesthesiologist VAS ratings were not significantly associated (OR 1.02, 95% CI 0.87–1.20).

In multivariable analysis adjusted for age, premedication, parental presence, and mode of induction, neither parental (aOR 1.13, 95% CI 0.95–1.36) nor anesthesiologist VAS ratings (aOR 1.02, 95% CI 0.84–1.24) were independently predictive of preoperative anxiety. However, younger age and inhalational induction remained significant predictors in both models (see Table 5).

**TABLE 5:** Multivariable logistic regression analysis of preoperative anxiety.

Variable	Parents			Anesthesiologists		
	aOR	95% CI	*p*	aOR	95% CI	*p*
VAS prediction	1.13	0.95–1.36	0.17	1.02	0.84–1.24	0.86
Premedication (yes)	0.67	0.19–2.32	0.52	0.77	0.22–2.71	0.67
Parental presence (yes)	1.85	0.50–6.93	0.35	2.29	0.66–8.25	0.19
Inhalation induction	3.88	1.31–13.08	0.019	3.67	1.27–12.01	0.022
Age (per year)	0.62	0.43–0.86	0.006	0.6	0.42–0.84	0.004

Abbreviations: aOR: adjusted odds ratio; VAS: Visual Analog Scale.

In subgroup analyses, parental predictions showed a moderate correlation with observed anxiety in children receiving premedication (*r*_s_ = 0.32, 95% CI −0.01–0.59) and in those undergoing inhalational induction (*r*_s_ = 0.33, 95% CI 0.03–0.58). Predictions by anesthesiologists were not significantly correlated with observed anxiety in any subgroup. The full set of subgroup results is presented in Table S2.

## 4 Discussion

This study aimed to evaluate how accurately parents and anesthesiologists can predict children's anxiety at anesthesia induction based on early impressions during routine preoperative consultations. Neither group was able to reliably anticipate a child's anxiety or compliance with anesthesia induction in advance. Children responding to a “high‐five” greeting showed lower anxiety levels, while the younger age of both child and parent and a migration background were associated with increased anxiety.

Our data align with the findings of MacLaren et al. [25], who reported limited predictive accuracy on the day of surgery. In our study, assessments took place earlier (as is common practice in Germany), allowing time to integrate the growing body of knowledge on predictors of perioperative anxiety. Involving parents in the prediction and management of anxiety is not only compassionate but also meaningful, as they are presumed to know their children best. That both parents and anesthesiologists struggle with prediction underscores the hospital environment as a major source of stress, often triggering unexpected anxiety in children. This suggests that current clinical practice, at least in this hospital, requires improvement to better support children through the preoperative process.

Interestingly, more than half of the parents in our cohort reported clinically significant levels of anxiety (VAS ≥ 4) at consultation. Although parental anxiety is associated with child distress [10, 17], it rarely resulted in clinical responses such as anxiolytic medication (as shown in Table 2). This indicates a disconnection between parental emotional state and clinical decision‐making. A similar mismatch was observed in parents' perceptions of the intervention: While most procedures were objectively classified as small or medium in extent, parents tended to rate them as both more extensive and more personally meaningful. Despite these discrepancies, observed preoperative anxiety did not differ across any of these categories in our study. Addressing parental anxiety and fostering family confidence should receive more attention, as it may indirectly improve the child's experience as shown in previous studies [17, 20, 32, 33].

Most clinicians performing anxiety predictions were residents. Limited pediatric experience may have contributed to the low predictive accuracy observed, highlighting the need for simple, pragmatic tools or observable behavioral cues to support early identification [13, 26]. Our study contributes to this effort by identifying low‐threshold indicators. A child's willingness to engage in a simple “high‐five” or handshake was associated with lower anxiety and may reflect resilience or social confidence—behaviors generally associated with lower anxiety. Since younger age was consistently linked to higher anxiety even within the narrow 2–6, we cannot exclude an age‐related bias, as younger children may have been less likely to respond to such a gesture. Nevertheless, the “high‐five” approach captures an aspect of social approach behavior that is familiar, non‐threatening, and child‐friendly. It is derived from clinical experience, where many pediatric anesthesiologists use such gestures to anticipate how a child may cope during preoperative preparation. Similar observations investigating children's behavior have shown that walking to the operating room is associated with less distress during anesthesia induction [34]. The “high‐five” approach in our study should therefore be viewed as an exploratory, child‐friendly behavioral cue that may hold promise and warrants further investigation in future studies.

The role of socioeconomic status (SES) remains inconsistently reported. Some studies found elevated anxiety in children from lower SES backgrounds [35, 36], while others did not [18, 37]. Our data support the latter. Broad healthcare access and strong social systems in Germany may buffer SES‐related disparities. However, children with a migration background showed higher anxiety levels, possibly due to language or cultural barriers, as similarly reported in Latino populations in the U.S. [38]. These children may represent a vulnerable group requiring targeted, culturally sensitive support.

Contrary to prior research, we found no association between parental and child anxiety [18, 26]. This may reflect methodological differences: previous studies used the STAI, while we employed the more pragmatic VAS. Still, reducing parental anxiety remains important, as it fosters a calmer environment and may serve as a quality marker [39]. The role of parental presence during induction is, however, complex. While calm, confident parents may help reduce a child's distress, anxious parents can amplify it [10]. Recent findings suggest an association between parental presence and more difficult induction behaviors, including poorer mask acceptance [13]. At the same time, many parents provide meaningful comfort, and parental presence is also a child's right [40]. Nonetheless, involving parents in a way that empowers them to provide emotional support and reassurance remains a key element of child‐centered perioperative care.

Lastly, IV induction was associated with lower observed anxiety than inhalational induction, contrary to earlier findings [41]. This likely reflects selection bias: IV access in our setting is typically achieved after topical anesthesia, while inhalational induction is more common in already distressed children, thus reflecting pre‐existing anxiety rather than the induction method itself.

Despite its pragmatic design, this study has several limitations. Children and families with language barriers were not included due to ethical requirements regarding the informed consent procedure. This represents a relevant limitation, as language barriers may be associated with higher preoperative anxiety and could therefore introduce a selection bias. As a compromise, the migration status was evaluated. As this study was conducted in a single tertiary hospital, the findings should be interpreted with caution and may not be directly generalizable to other healthcare settings. Perioperative practices such as premedication and parental presence, as well as interactions with perioperative staff, can vary across institutions and countries and may influence anxiety levels and observed associations. These effects may be even more pronounced in specialized pediatric centers with greater routine and experience in caring for young children. Anxiety was assessed using the observational mYPAS‐SF supplemented by the ICC. The act of inquiring about anxiety may itself have influenced distress levels. However, both children and parents had the opportunity to discuss concerns with the study team or clinical staff at any time. The study setting was heterogeneous with respect to key factors known to affect perioperative anxiety, such as the use of premedication, parental presence, and mode of induction. In our multivariable analyses, certain contextual factors also showed a stronger association with children's anxiety than the predictive ratings themselves. This underscores the multifactorial nature of perioperative distress and the need to account for these influences when interpreting predictive assessments. Although this observational study does not meet the methodological rigor required to provide a definitive solution for addressing this complexity, our findings offer a valuable first step toward understanding how simple, pragmatic assessments might be meaningfully integrated into everyday perioperative care. In the future, broader positively framed outcome domains including satisfaction, perceived autonomy, and postoperative recovery could provide a more comprehensive picture of perioperative well‐being [42, 43]. The anesthesiologists conducting the preoperative assessments were not involved in the induction and were unaware of the specific team performing anesthesia induction. While this may have limited predictive accuracy by excluding team‐related factors, it also reflects real‐world clinical conditions. Nevertheless, all teams were expected to provide child‐appropriate and attentive care in line with institutional standards. Although the VAS is not a validated tool for assessing preoperative anxiety in young children, it has been pragmatically used in similar perioperative settings across a wide pediatric age range (2–16 years) to predict children's anxiety [5]. Previous studies have demonstrated strong correlations between parental and anesthesiologist VAS ratings and observational anxiety scores obtained with the mYPAS (*r* = 0.68 and *r* = 0.73, respectively) [7]. Objective markers such as heart rate variability or cortisol were not assessed. Future studies should consider combining behavioral measures with physiological indicators to improve and confirm predictive validity [20, 44].

Our findings have two main clinical and scientific implications. First, anxiety management should be viewed as a dynamic and ongoing process rather than a single‐point intervention. Individualized strategies are needed to identify and manage preoperative anxiety in young children. Simple behavioral cues, such as a child's willingness to engage socially, may support early identification of children with lower anxiety levels. Future studies should explore whether reliably identifying such children could allow for more selective use of pharmacological interventions. Second, the predictive accuracy of anxiety assessments may serve as a pragmatic quality indicator in pediatric anesthesia. As patient‐reported outcome measures (PROMs) gain importance in pediatric anesthesia [39, 42, 43], prediction metrics could reflect how effectively child‐centered and anxiety‐reducing strategies are implemented in clinical routine, making accurate prediction a potential marker for the quality of pediatric anesthesia care.

Parental and anesthesiologists' predictions at an early stage are limited, so incorporating simple behavioral cues into routine practice and incorporating family's context could enhance early recognition of preoperative anxiety and support more efficient, child‐centered decision‐making.

## Author Contributions

A.S.: Conceptualization, Methodology, Validation, Formal analysis, Investigation, Resources, Data curation, Visualization, Writing – Original Draft, Writing – Review and Editing, Project administration. M.G., C.N., A.K., K.K.: Conceptualization, Formal analysis, Investigation, Resources, Data curation, Writing – Review and Editing. H.S., A.B.: Formal analysis, Writing – Original Draft, Writing – Review and Editing. T.B.: Conceptualization, Methodology, Validation, Formal analysis, Writing – Original Draft, Writing – Review and Editing, Project administration.

## Funding

The authors have nothing to report.

## Ethics Statement

This trial was approved by the local Institutional Review Board (Ethics Committee of Christian‐Albrechts‐University of Kiel, Germany, ethics approval number D508/24) and performed in accordance with the relevant guidelines and regulations as well as in accordance with the Declaration of Helsinki.

## Consent

Informed consent was obtained from all parents or legal guardians of participants involved in the study.

## Conflicts of Interest

The authors declare no conflicts of interest.

## Supporting information


**Figure S1:** Distribution of children's responses to the “high‐five” test across age groups.

**Table S1:** Applied non‐pharmacological.

**Table S2:** Subgroup analyses of correlations between predictions and mYPAS‐SF scores.

## Data Availability

The authors confirm that the data supporting the findings of this study are available within the article [and/or] its Supporting Information.
