# Supplementary figures and images for: Predicting Anxiety in Children Aged 2–6 During Preoperative Anesthesia Consultation—A Prospective Observational Study
Source: Paediatr Anaesth. 2025 Dec 9;36(3):281–91. doi: 10.1002/pan.70101 (PMC12887142; doi:10.1002/pan.70101)

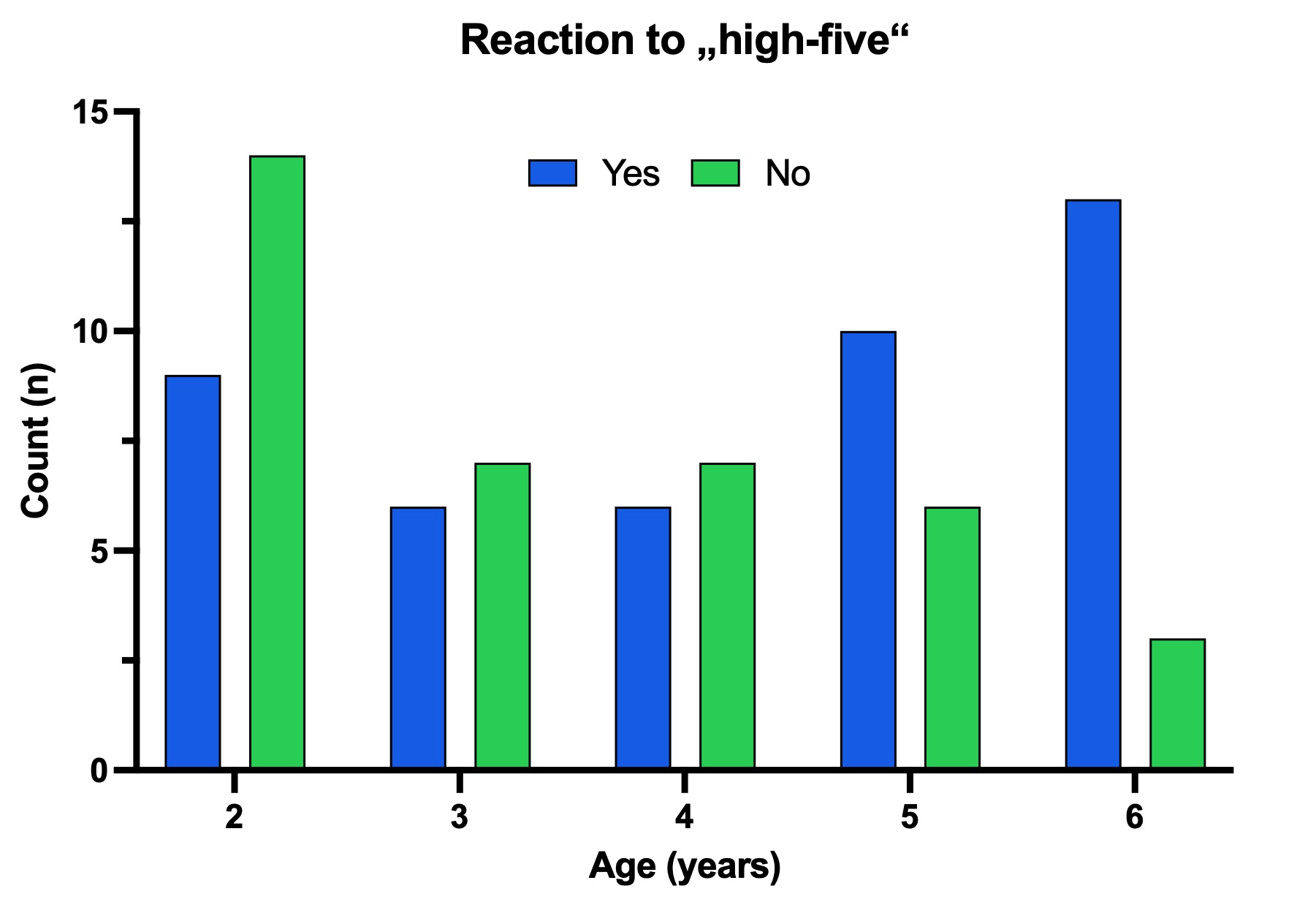

Supplement: Supplementary file 1 — Figure S1: Distribution of children's responses to the “high‐five” test across age groups. [file PAN-36-281-s002.jpg]
