# Supplementary material for: Predicting Anxiety in Children Aged 2–6 During Preoperative Anesthesia Consultation—A Prospective Observational Study
Source: Paediatr Anaesth. 2025 Dec 9;36(3):281–91. doi: 10.1002/pan.70101 (PMC12887142; doi:10.1002/pan.70101)
Supplement: Supplementary file 2 — Table S1: Applied non‐pharmacological. [file PAN-36-281-s001.docx]

Supplentary Table 1 Applied non-pharmacological

| **Intervention** | **Total N=149** |
| --- | --- |
| Parental presence | 62 (41.6%) |
| Music | 24 (16.1%) |
| Suggestion/distraction | 17 (11.4%) |
| Clown | 17 (11.4%) |
| Other | 14 (9.4%) |
| Video presentation | 8 (5.4%) |
| Toy/stuffed animal | 7 (4.7%) |

Values are presented as number (proportion). Multiple answers were possible.
