# Supplementary material for: Predicting Anxiety in Children Aged 2–6 During Preoperative Anesthesia Consultation—A Prospective Observational Study
Source: Paediatr Anaesth. 2025 Dec 9;36(3):281–91. doi: 10.1002/pan.70101 (PMC12887142; doi:10.1002/pan.70101)
Supplement: Supplementary file 3 — Table S2: Subgroup analyses of correlations between predictions and mYPAS‐SF scores. [file PAN-36-281-s003.docx]

Supplementary Table 3 *Subgroup analyses of correlations between predictions and mYPAS-SF scores.*

| **Subgroup** |  | **Parental prediction** | | **Anesthesiologists prediction** | |
| --- | --- | --- | --- | --- | --- |
|  | **n** | **r_s_ (95% CI)** | **P-value** | **r_s_ (95% CI)** | **P-value** |
| Use of premedication |  |  |  |  |  |
| Yes | 38 | 0.32 (-0.01 to 0.59) | 0.047 | 0.29 (-0.04 to 0.57) | 0.073 |
| No | 54 | 0.19 (-0.09 to 0.44) | 0.168 | 0.07 (-0.21 to 0.33) | 0.638 |
| Anesthesia Induction |  |  |  |  |  |
| Intravenous | 47 | 0.07 (-0.23 to 0.35) | 0.657 | -0.07 (-0.36 to 0.23) | 0.637 |
| Inhalation | 45 | 0.33 (0.03 to 0.58) | 0.026 | 0.24 (-0.07 to 0.50) | 0.114 |
| PPIA |  |  |  |  |  |
| Yes | 66 | 0.15 (-0.10 to 0.39) | 0.218 | 0.03 (-0.22 to 0.28) | 0.801 |
| No | 26 | 0.32 (-0.09 to 0.63) | 0.114 | 0.31 (-0.10 to 0.63) | 0.125 |

Abbreviations: mYPAS-SF, Short Form of the Modified Yale Preoperative Anxiety Scale; PPIA: Parental Presence during induction of anesthesia
